# Antibodies to the Extracellular Pore Loop of TRPM8 Act as Antagonists of Channel Activation

**DOI:** 10.1371/journal.pone.0107151

**Published:** 2014-09-09

**Authors:** Silke Miller, Sara Rao, Weiya Wang, Hantao Liu, Judy Wang, Narender R. Gavva

**Affiliations:** Department of Neuroscience, Amgen Inc., Thousand Oaks, California, United States of America; University of South California, United States of America

## Abstract

The mammalian transient receptor potential melastatin channel 8 (TRPM8) is highly expressed in trigeminal and dorsal root ganglia. TRPM8 is activated by cold temperature or compounds that cause a cooling sensation, such as menthol or icilin. TRPM8 may play a role in cold hypersensitivity and hyperalgesia in various pain syndromes. Therefore, TRPM8 antagonists are pursued as therapeutics. In this study we explored the feasibility of blocking TRPM8 activation with antibodies. We report the functional characterization of a rabbit polyclonal antibody, ACC-049, directed against the third extracellular loop near the pore region of the human TRPM8 channel. ACC-049 acted as a full antagonist at recombinantly expressed human and rodent TRPM8 channels in cell based agonist-induced ^45^Ca^2+^ uptake assays. Further, several poly-and monoclonal antibodies that recognize the same region also blocked icilin activation of not only recombinantly expressed TRPM8, but also endogenous TRPM8 expressed in rat dorsal root ganglion neurons revealing the feasibility of generating monoclonal antibody antagonists. We conclude that antagonist antibodies are valuable tools to investigate TRPM8 function and may ultimately pave the way for development of therapeutic antibodies.

## Introduction

The transient receptor potential melastatin 8 (TRPM8) channel is a non-selective cation channel that is activated by cold temperature (below ∼23°C) or compounds that cause a cooling sensation, such as menthol and icilin [1]–[3]. TRPM8 is expressed in a subpopulation of small to medium diameter neurons in the trigeminal and dorsal root ganglia that confer sensitivity to innocuous cold in the somatosensory system [4]. Three independently generated mouse models lacking functional TRPM8 channels exhibit attenuated cold sensation at a discrete temperature range in behavioral assays [5]–[7]. TRPM8 channels not only mediate behavioral, but also autonomic responses to innocuous cold, including the regulation of body temperature [8]–[10] and potentially cutaneous vascular tone [11]. Supporting these findings, TRPM8 expression was reported in other tissues, including the respiratory tract, urinary system, and vasculature [11], [12]. Thus, TRPM8 may play multiple functional roles, likely to be in a tissue-dependent manner, not only under innocuous conditions, but also in disease states. Cold hypersensitivity and hyperalgesia are symptoms of several neuropathic conditions [13], including painful bladder syndrome [14], and chemotherapy-induced neuropathy [15]. Genetic ablation of TRPM8 in mice abolishes cold-evoked behaviors after peripheral inflammation or nerve injury [6] and in models of chemotherapy-induced neuropathy [16]. Similarly, selective ablation of TRPM8 positive neurons in mice results in reduced sensitivity to innocuous cold, attenuated cold hypersensitivity and loss of cooling-mediated analgesia after injury [17]. Lastly, small molecule antagonists are efficacious in animal models of neuropathy [18] and overactive bladder [19], thus supporting a potential therapeutic benefit of TRPM8 antagonists.

As an alternative to small molecules, antibodies that bind near the pore regions of ion channels have been shown to antagonize channel activation [20]–[22]. Antibodies are known to exhibit exquisite specificity and unlimited diversity and could therefore provide advantages over small molecules. Due to their long plasma half-life, antibodies may represent better therapeutic agents for chronic disease conditions, including neuropathic pain. In addition, antibodies are generally peripherally restricted and therefore devoid of central side-effects.

To explore the possibility to target TRPM8 with antagonist antibodies, we have characterized commercially available poly- and monoclonal antibodies directed against the pore loop of TRPM8 as antagonists of cold as well as chemical ligand activation.

## Materials and Methods

### Reagents

**Figure 1:**
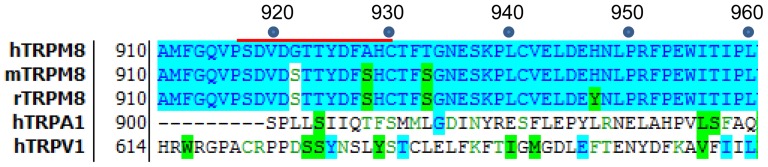
Alignment of extracellular pore loop sequences. Alignment of the third extracellular loop sequences of the human, rat and mouse TRPM8 channel and the human TRPA1 and TRPV1 channels. The red line indicates the epitope sequence that ACC-049 was generated against.

TRPM8 positive control antagonist, compound M8-B [9], TRPV1 positive control antagonist, AMG6451 [23], and TRPA1 positive control antagonist, AMG9090 [24] all were synthesized at Amgen, Inc. A list of the antibodies used is shown in Table 1 and the amino acid homology of the third extracellular loop of different TRP channels is shown in Figure 1. ACC-049, a rabbit polyclonal TRPM8 antibody generated against an epitope in the third extracellular loop near the pore region of human TRPM8 was purchased from Alomone labs (Jerusalem, Israel). Its cognate peptide (SDVD GTTYDFAHC corresponding to amino acid residues 917-929 of human TRPM8) was also purchased from Alomone labs. Other rabbit polyclonal antibodies generated against the third extracellular loop near the pore region were purchased from Thermo Scientific (Waltham, MA), Antibodies online (Atlanta, GA) and Enzo Lifesciences (Farmingdale, NY). Rabbit monoclonal antibodies generated against the third extracellular loop near the pore region were purchased from MyBiosource (San Diego, CA), Creative Diagnostics (Shirley, NY) and Lifespan Biosciences (Seattle, WA). Reagents used in the study were purchased from the following companies: Icilin and menthol were purchased from Sigma-Aldrich (St. Louis, MO). Ham's F-12 nutrient mixture, DMEM, 1x glutamine-penicillin-streptomycin, 1x non-essential amino acids, dialyzed fetal bovine serum, genetecin, blasticidin-S-HCl, zeocin; Alexa fluor 488 and Hoechst 33342 were purchased from Invitrogen (Carlsbad, CA). Tetracycline-free fetal bovine serum was purchased from Hyclone (Logan, UT), Tetracycline hydrochloride from Cellgro Mediatech Inc. (Hemdon, VA). Neurobasal medium with 1X B-27 Supplement and 1X Glutamax was purchased from Life technologies (Grand Island, NY), Insulin was from Sigma (St. Louis, MO). Cytostar-T plates, poly-_D_-lysine coated Cytostar-T plates, calcium-45-radionuclide (^45^Ca^2+^) and microplate scintillation counter TopCount NXT-Packard were purchased from PerkinElmer life and analytical sciences (Waltham, MA). Control IgG was purchased from Jackson ImmunoResearch Laboratories, Inc. (Westgrove, PA). Fatal-Plus Solution was purchased from Vortech Pharmaceuticals (Dearborn, MI), the Papain Dissociation System kit from Worthington Biochemical Corp. (Lakewood, NJ)

**Table 1:** Source and characteristics of the antibodies.

Source	Catalog number	Lot number(s)	Clonality	Epitope lies within
Alomone labs	ACC-049	AN0010, 0011, 0012	Polyclonal	917–929
MyBioSource	MBS609041	13100131	Monoclonal	925–975
Creative Diagnostics	CDB890	CABT37242RH	Monoclonal	920–960
Thermo Scientific	OST00133W	PB1818654	Polyclonal	900–950
Antibodies Online	ABIN351226	Rb0496-290608W	Polyclonal	900–950
Lifespan Biosciences	LS-B6668	53021	Monoclonal	900–1000
EnzoLifesciences	BML-SA664	02261432	Polyclonal	917–929

Except for ACC-049, the antigen sequence used for the antibodies was not disclosed by the vendors.

### Cell-based assays

Complementary DNAs of recombinant human, rat and mouse TRPM8 were stably transfected into Tetracycline-Regulated Expression cell line, T-REx-Chinese hamster ovary cell line from Invitrogen, Inc. (Carlsbad, CA). Twenty four hours before the assay, cells were seeded in Cytostar-T 96-well plates at a density of 3.0×10^4^ per well, and TRPM8 channel expression was induced with 0.5 µg/ml tetracycline. On the day of the assay, culture media were removed, and cells were incubated with TRPM8 antibody (ACC-049), antagonist M8-B, or control IgG for 30 min before addition of ^45^Ca^2+^. Stock solutions of ACC-049, M8-B, and control IgG were diluted to the desired final concentrations in the assay buffer (F-12 Ham's containing 15 mM HEPES and 0.1 mg/ml BSA) keeping the final concentration of DMSO lower than 0.5% for M8-B. The activity of TRPM8 was monitored as function of cellular uptake of radioactive calcium. The final concentration of ^45^Ca^2+^in the ^45^Ca^2+^ uptake assay was 10 µCi/ml. Icilin (1 µM) or menthol (100 µM) induced ^45^Ca^2+^ uptake was used for evaluating blockade of chemically induced TRPM8 channel activation by ACC-049, antagonist M8-B and control IgG, as reported previously [25]. For cold activation of TRPM8, cold assay buffer at 10°C was used. ACC-049 was assayed in cells stably expressing human, rat, or mouse TRPM8 channels. The other commercial antibodies listed in Table 1 were assayed at a single concentration in 30 µl in icilin induced activation of human TRPM8 channels. The TRPM8 antagonist M8-B was used as a positive control and control IgG was used as a negative control in all assays. TRPM8 activation was calculated as percent of control (POC) with agonist (cold, icilin or menthol) induced radioactive calcium uptake set as 100 percent and assay buffer only plus ^45^Ca^2+^ set as zero percent with exception of cold activation, where blockade with M8-B at 1 µM was set as zero percent. Evaluation of antagonism at heat activation of TRPV1 [26], and noxious cold temperature activation of TRPA1 [24] was conducted as reported previously.

### Dorsal root ganglion (DRG) neuron preparation

Six male Sprague Dawley rats weighing 150–200 g were obtained from Harlan (Hayward, CA). Rats were cared for in accordance to the *Guide for the Care and Use of Laboratory Animals, 8^th^* Edition [27] and research protocols were approved by Amgen's Institutional Animal Care and Use Committee. Rats were group housed in facilities internationally accredited by the Association for the Assessment and Accreditation of Laboratory Animal Care (AALAC). Rats were kept in non-sterile ventilated micro-isolator housing on corn cob bedding. Rats had *ad libitum* access to pelleted feed (Harlan Teklad 2020X, Indianapolis, IN) and water (on site reverse osmosis-purified) via automatic watering system or water bottle. Rats were maintained on a 12∶12 hour light-dark cycle in rooms at (70±5°F, 50±20% RH) and had access to enrichment opportunities. On the experimental day, rats were sacrificed by an overdose of Fatal-Plus Solution and approximately 20 DRGs per animal were removed. DRG neurons were isolated from the ganglia using the Papain Dissociation System kit according to the manufacturer's instructions. Pelleted neurons were then immediately resuspended in Neurobasal medium with 1X B-27 Supplement and 1X Glutamax, containing 10 µg/ml of Insulin. Neurons were then seeded in poly-_D_-lysine coated Cytostar-T 96 well plates at a density of 2.0×10^4^ per well and incubated at 37°C in a humidified atmosphere of 5% CO_2_ for 24 hours. On the day of the assay, culture media were removed and neurons were subjected to the ^45^Ca^2+^ uptake assay as described above.

### Statistical Analysis

GraphPad Prism 5.0 (GraphPad Software, Inc., San Diego, CA) software was used to plot the data and to calculate the 50% inhibitory concentrations (IC_50_) of compounds and antibodies using a sigmoidal dose-response equation.

## Results

We have evaluated the ability of polyclonal antibody ACC-049 that was generated against the pore loop of TRPM8 to act as an antagonist in a functional cell based ^45^Ca^2+^ uptake assay using icilin, menthol, or cold buffer as agonists in cultured Chinese hamster ovary (CHO) cells constitutively expressing human, rat or mouse TRPM8 channels, as well as in dissociated rat dorsal root ganglion neurons endogenously expressing rat TRPM8 channels.

### ACC-049 acts as a selective antagonist of TRPM8

**Figure 2:**
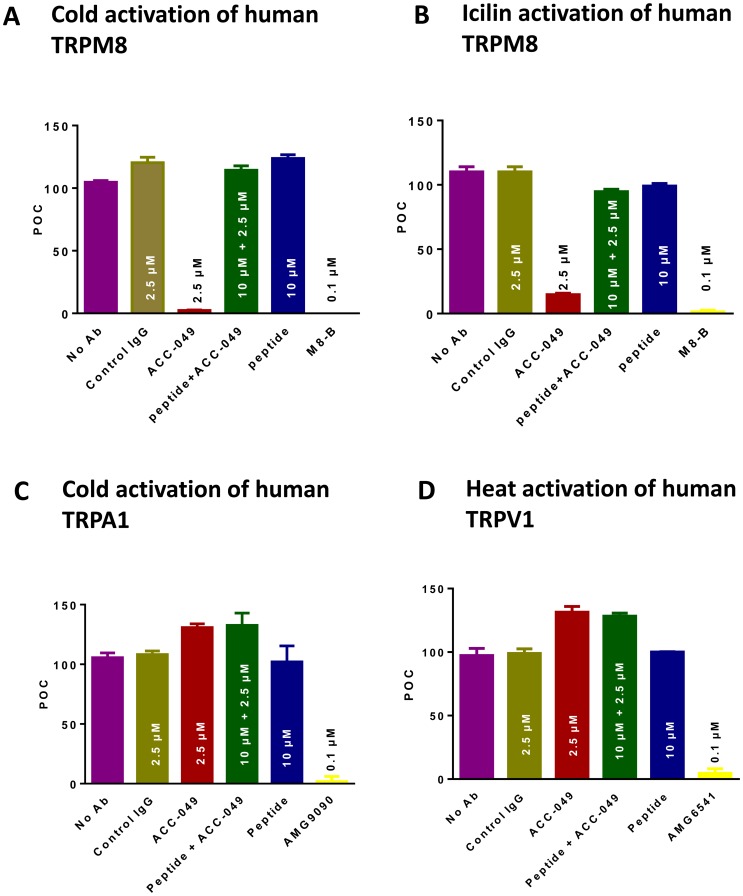
ACC-049 is a selective inhibitor of TRPM8. Specificity of ACC-049 (2.5 µM) for blocking human TRPM8 activation induced by the specific natural agonist cold (A) or synthetic agonist icilin (D). No effect of ACC-049 on noxious cold induced human TRPA1 (B) or heat induced TRPV1 activation (C). Small molecule antagonists AMG9090 and AMG6541 are the positive control for TRPA1 (B) or TRPV1 (C) blockage, respectively. Note the near complete blockade of TRPM8 activation by ACC-049 at 2.5 µM, similar to that by the positive small molecule antagonist control M8-B (A). Neither control IgG, nor peptide-absorbed ACC-049, or peptide alone blocked activation of any of the channels tested (A–D). Values are means of triplicate measures in a single experiment and expressed as percent of control (POC). Agonist induced ^45^Ca^2+^ uptake in the absence of antibodies (no Ab) was considered as 100 percent and wells with small molecule antagonists plus ^45^Ca^2+^ were set as zero percent.

In a single concentration (2.5 µM) evaluation, ACC-049 specifically inhibited activation of human TRPM8 by cold temperature (Fig. 2A) or synthetic agonist icilin (Fig. 2B), but had no effects on noxious cold temperature activation of human TRPA1 (Fig. 2C) or heat activation of human TRPV1 (Fig. 2D). ACC-049 blocked cold activation of human TRPM8 nearly completely (Fig. 2A) and icilin activation by more than 80% (Fig. 2B). The same concentration (2.5 µM) of control IgG had no effect on TRPM8, TRPA1 or TRPV1 activation (Fig. 2A–D). Pre-absorption with an excess amount (10 µM) of the peptide that ACC-049 was generated against abolished the antagonism of cold (Fig. 2A) and icilin (Fig. 2B) activation of human TRPM8 channels, whereas no effects were observed on TRPA1 (Fig. 2C) or TRPV1 (Fig. 2D) channel activation. The peptide alone had no effects on TRPA1, TRPM8, or TRPV1 activation (Fig. 2A–D). The positive control compounds, M8-B (Fig. 2A, B), AMG9090 (Fig. 2C), and AMG6451 (Fig. 2D), completely inhibited activation of human TRPM8, human TRPV1 and human TRPA1, respectively.

### ACC-049 blocks cold activation of human and rodent TRPM8 channels in a concentration dependent manner

**Figure 3:**
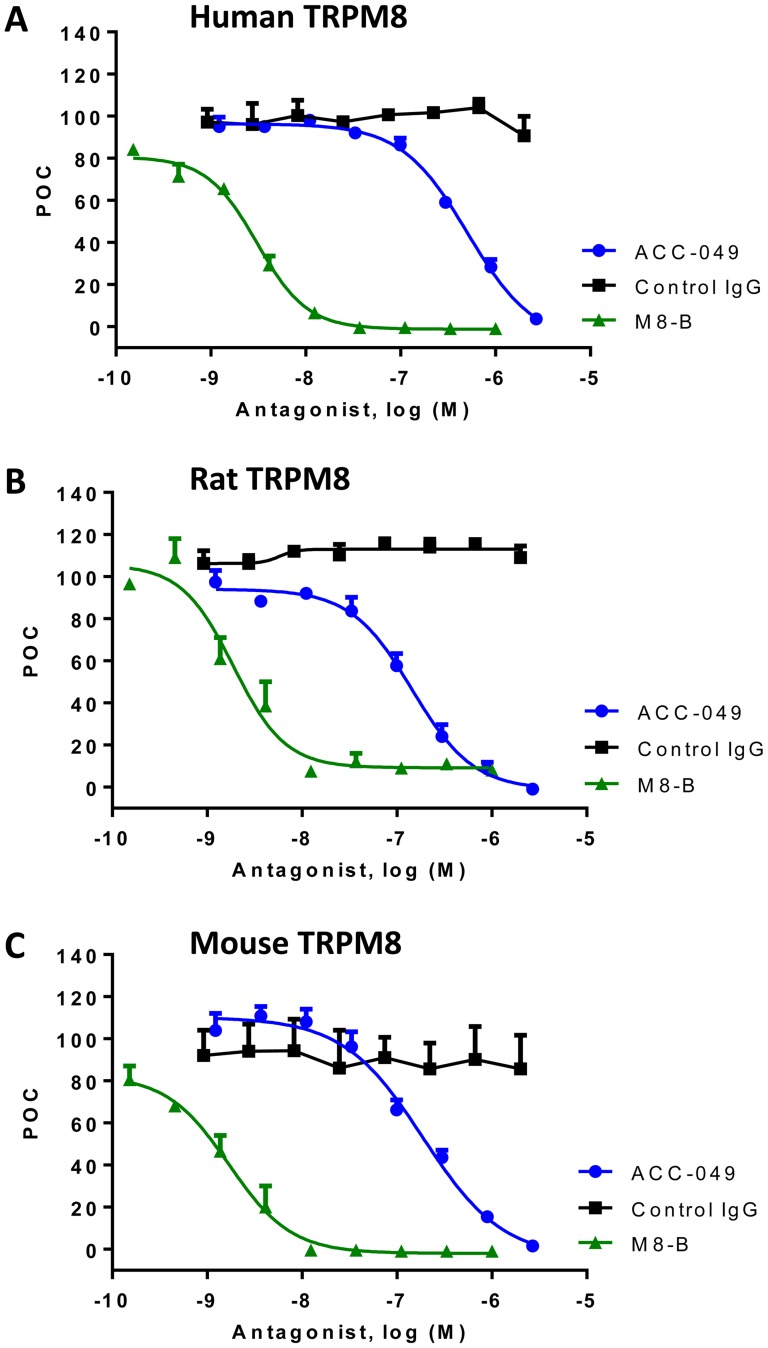
Cold activation of TRPM8. Concentration dependent antagonism of cold activation (10°C) of the human (A), rat (B), or mouse (C) TRPM8 channels by ACC-049, control IgG and M8-B measured by ^45^calcium uptake. Note the right shifted concentration response of ACC-049 on human TRPM8 (A) compared to rat (B) or mouse (C) TRPM8, while the small molecule antagonist positive control M8-B exhibited comparable responses on TRPM8 channels of all species tested (A–C). Values are means of triplicate measures in a single experiment and expressed as percent of control (POC). Cold induced ^45^Ca^2+^ uptake was considered as 100 percent and wells with M8-B at 1 µM plus ^45^Ca^2+^ were set as zero percent.

In order to determine the potency of ACC-049 at cold temperature (10°C) activation of human and rodent TRPM8, various concentrations of ACC-049 were incubated with CHO cells expressing human, rat or mouse TRPM8 channels prior to measuring cold-induced ^45^Ca^2+^ uptake. ACC-049 showed a concentration dependent inhibition of TRPM8 activation by cold (Fig. 3 and Table 2). ACC-049 inhibited cold activation of human TRPM8 channels with an IC_50_ value of 508±1.2 nM, and was slightly more potent at blocking rat and mouse channels with IC_50_ values of 144±1.2 nM and 188±1.3 nM, respectively (Fig. 3A–C and Table 2). Positive control small molecule antagonist M8-B inhibited human and rodent TRPM8 with IC_50_ values ranging from 2 to 4 nM, the same range as previously reported [9], while the negative control IgG had no effects (Table 2).

**Table 2:** IC_50_ values (nM) of cold, icilin, and menthol induced human, rat, or mouse TRPM8 channel activation by ACC-049.

	Human TRPM8 (nM)	Human TRPM8 (nM)	Rat TRPM8 (nM)	Rat TRPM8 (nM)	Mouse TRPM8 (nM)	Mouse TRPM8 (nM)
	ACC-049	M8-B	ACC-049	M8-B	ACC-049	M8-B
Cold	508±1.2	3±1.1	144±1.2	2±1.2	188±1.3	2±1.2
Icilin	1054±1.3	11±1.2	252±1.1	11±1.1	363±1.2	6±1.1
Menthol	1160±1.1	1±1.4	>2000	2±1.3	>2000	2±1.2

Values are expressed as mean ± SD of one experiment conducted in triplicate wells in a 96 well format. Concentration ranges of ACC-049 and respective agonists are described in the text. IC_50_ of control IgG in all assays was >2500 nM.

### ACC-049 blocks icilin activation of human and rodent TRPM8 channels in a concentration dependent manner

**Figure 4:**
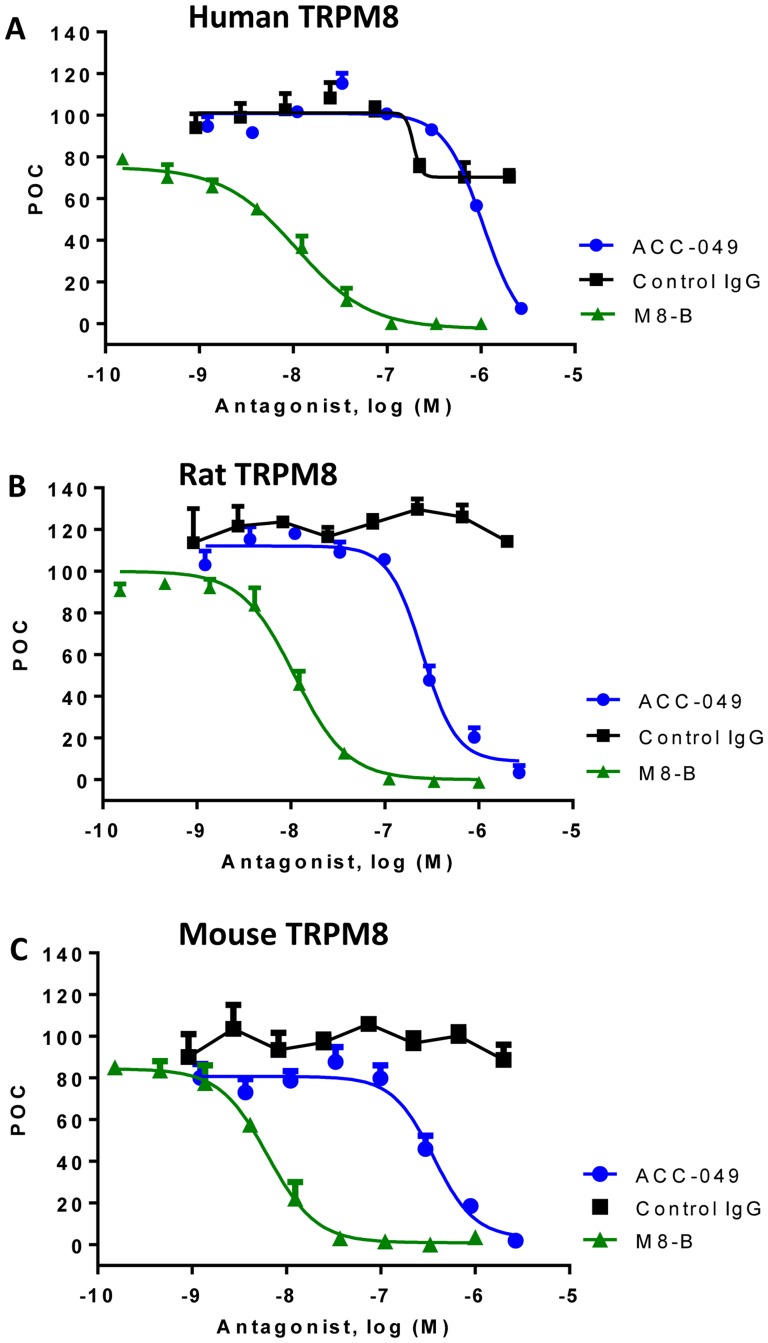
Icilin activation of TRPM8. Concentration dependent antagonism of icilin induced activation of the human (A), rat (B) or mouse (C) TRPM8 channels by ACC-049, control IgG and M8-B measured by ^45^calcium uptake. Note the right shifted concentration response of ACC-049 on human TRPM8 (A) compared to rat (B) or mouse (C) TRPM8, while the small molecule antagonist positive control M8-B exhibited comparable responses on TRPM8 channels of all species tested (A–C). Values are means of triplicate measures in a single experiment and expressed as percent of control (POC). Icilin induced ^45^Ca^2+^ uptake was considered as 100 percent and wells with only assay buffer plus ^45^Ca^2+^ were set as zero percent.

Next, we determined the potency of ACC-049 at icilin activation of human and rodent TRPM8 by preincubation of different concentrations of ACC-049 prior to quantitation of icilin-induced ^45^Ca^2+^ uptake. Similar to the blockade of cold activation of TRPM8, ACC-049 inhibited activation of rat and mouse TRPM8 channels by 1 µM icilin more potently than human TRPM8 with respective IC_50_ values of 252±1 nM and 363±1 nM, as compared to human TRPM8 channels with an IC_50_ value of 1054±1 nM (Fig. 4A–C and Table 2). Positive control small molecule antagonist M8-B inhibited human and rodent TRPM8 channels with IC_50_ values ranging from 6 to 11 nM, the same range as previously reported [9], while the negative control IgG had no effects (Table 2).

### Species differences in ACC-049 inhibition of TRPM8 by menthol activation

**Figure 5:**
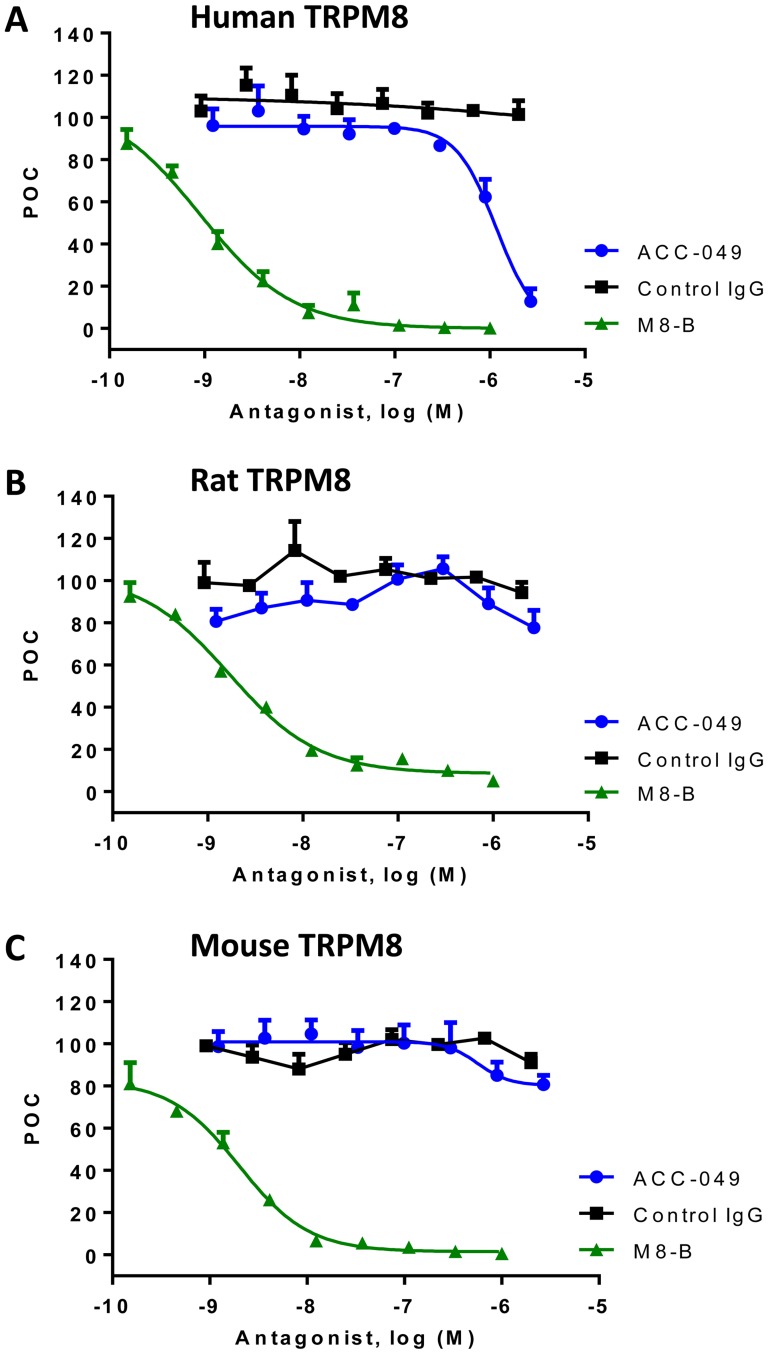
Menthol activation of TRPM8. Concentration dependent antagonism of menthol induced activation of the human (A), rat (B) or mouse (C) TRPM8 channels by ACC-049, control IgG and M8-B measured by ^45^calcium uptake. Human TRPM8 channel activation was blocked by ACC-049 in a concentration dependent manner (A), but there was no antagonistic effect of ACC-049 on either rat (B) or mouse (C) TRPM8 channels activated by menthol. The small molecule antagonist positive control M8-B exhibited comparable responses on TRPM8 channels of all species tested (A–C). Values are means of triplicate measures and expressed as percent of control (POC). Menthol induced ^45^Ca^2+^ uptake was considered as 100 percent and wells with only assay buffer plus ^45^Ca^2+^ were set as zero percent.

ACC-049 showed concentration dependent antagonism of human but not rodent TRPM8 activation by menthol revealing species differences. IC_50_ value at TRPM8 activation by 100 µM menthol was 1160±1 nM at human TRPM8 whereas no inhibition was observed up to 2 µM concentration of ACC-049 in CHO cells expressing either rat or mouse TRPM8 (Fig. 5A-C and Table 2). The positive control M8-B inhibited TRPM8 activation with similar potency (IC_50_ values of 1 to 2 nM) at all three species and the negative control IgG had no effects (Fig. 3–5 and Table 2).

### Antagonism of poly-and monoclonal antibodies against the third extracellular loop

**Figure 6:**
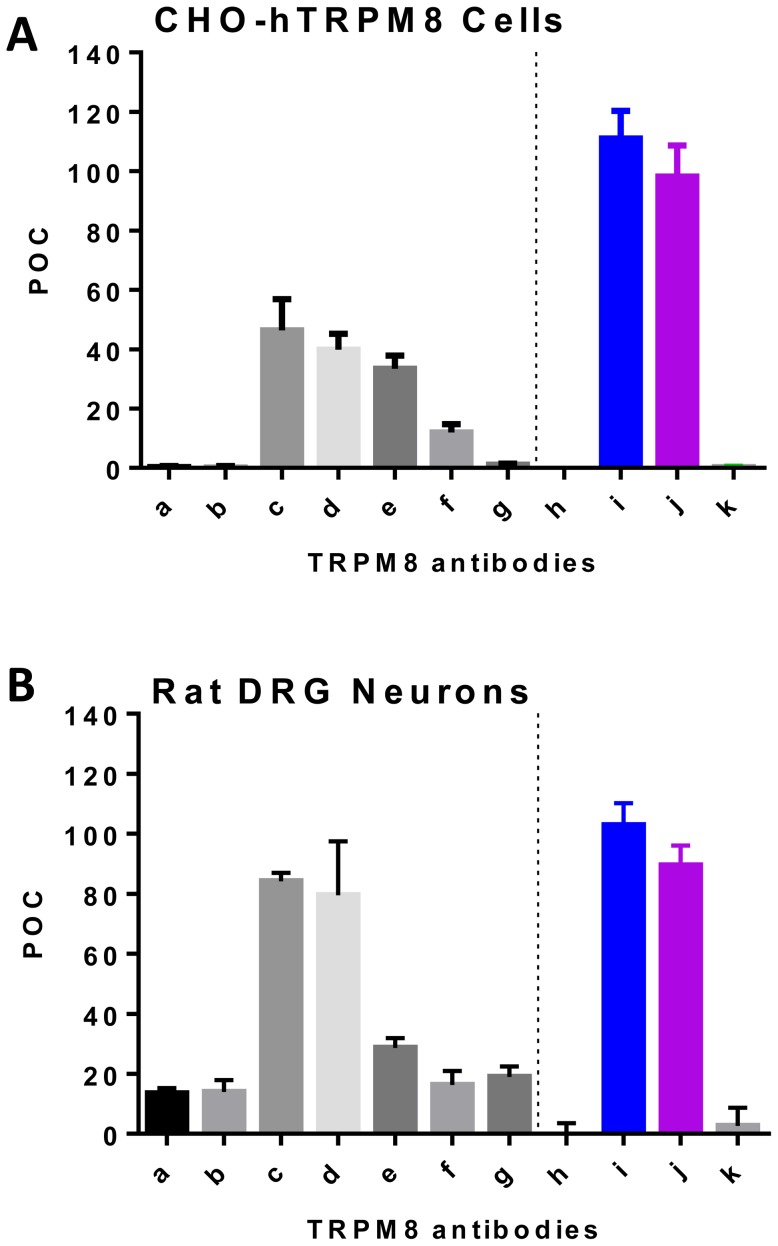
Icilin activation of TRPM8 in CHO cells and rat DRG neurons. Antagonism of icilin induced activation of human TRPM8 recombinantly expressed by CHO cells (A) and rat DRG neurons (B) by additional poly- and monoclonal antibodies generated against the third extracellular pore loop. **a**. Alomone ACC-049. **b**. MyBiosource MBS609041. **c**. Creative Diagnostics CABT37242RH. d. Thermo Scientific OST00133W. **e**. Antibodies Online ABIN351226. **f**. Lifespan Biosciences LS-B6668. **g**. Enzo Lifesciences BML-SA664. **h**. M8-B. **i**. 1 µM icilin. **j**. 1 µM icilin + peptide (SDVD GTTYDFAHC). **k**. Buffer. A. Note the complete block of TRPM8 channel activation by ACC-049 (**a**), MyBiosource (**b**) and Enzo Lifesciences (**g**) antibodies at the single concentration tested. Small molecule positive control M8-B also completely blocked TRPM8 channel activation (**h**). B. Five out of seven antibodies tested (**a, b, e, f, g**) block icilin activation of rat DRG neurons by 70–80%, two antibodies (**c, d**) are ineffective. Values are means of triplicate measures in a single experiment and expressed as percent of control (POC). ^45^Ca^2+^ uptake of CHO-TRPM8 cells activated with 1 µM icilin and antigen peptide (**j**) was considered as 100 percent and wells with only assay buffer plus ^45^Ca^2+^ were set as zero percent.

To further confirm that the blocking capabilities of ACC-049 were due to its binding to the extracellular pore loop, we tested additional six commercially available antibodies (Table 1) that were also generated against the third extracellular loop (the pore region) of human TRPM8 at a single concentration/volume in an icilin-induced ^45^Ca^2+^ uptake assay. All antibodies, polyclonal and monoclonal, blocked TRPM8 activation by 60% to 100% inhibition (Fig. 6). These data can only be interpreted in a qualitative manner because the concentrations of the antibody stocks were not provided by the manufacturers. Therefore, the differences seen in potency could be simply due to the differences of antibody concentration in the 30 µl of antibody solution used for the assay. At this single concentration, both poly- and monoclonal antibodies significantly blocked TRPM8 activation in this assay. Two polyclonal antibodies, ACC-049 (Alomone) and BML-SA664 (Enzo Life Sciences) as well as one monoclonal antibody, MBS609041 (MyBiosource) blocked icilin-induced ^45^Ca^2+^ uptake fully, as did the positive control antagonist M8-B. Other monoclonal (CABT 37242RH from Creative Diagnostics; LS-B6668 from Lifespan Biosciences) and polyclonal (OST00133W from Thermo Scientific; ABIN351226 from Antibodies online) antibodies blocked icilin-induced ^45^Ca^2+^ uptake partially. The peptide corresponding to the epitope in ACC-049 had no effects on its own in the icilin-induced ^45^Ca^2+^ uptake assay.

In order to test the ability of antibodies to block native TRPM8 channels, we repeated the icilin-induced ^45^Ca^2+^ uptake assay with the same concentration/volume of the antibodies using freshly isolated rat DRG neurons. ACC-049 (Alomone), MBS609041 (MyBiosource), LS-B6668 (Lifespan Biosciences),BML-SA664 (Enzo Life Sciences), and ABIN351226 (Antibodies online) blocked icilin-induced ^45^Ca^2+^ uptake by 70 - 80% and CABT 37242RH (Creative Diagnostics) and OST00133W (Thermo Scientific) did not show significant block of icilin-induced ^45^Ca^2+^ uptake in this assay. Buffer alone or the peptide corresponding to the epitope recognized by ACC-049 did not affect the magnitude of icilin-induced on ^45^Ca^2+^ uptake. The positive control antagonist M8-B showed full inhibition of icilin-induced ^45^Ca^2+^ uptake in rat DRG neurons.

## Discussion

In order to assess if antibodies could block TRPM8 channels, we have first characterized a commercially available rabbit polyclonal antibody (ACC-049) as a TRPM8 selective antagonist using agonist-induced cell-based ^45^Ca^2+^ uptake assays. This antibody acted as a full antagonist of TRPM8 activation by cold and icilin but showed species-specific differences at menthol activation.

ACC-049 blocked activation of human TRPM8 expressed in CHO cells by all three agonists tested, i.e., cold, icilin, and menthol. In contrast, only cold and icilin, but not menthol-induced activation was blocked by ACC-049 in rat and mouse TRPM8 expressing CHO cells. Interestingly, ACC-049 was approximately 2 to 4 fold more potent on rodent than human TRPM8 in blocking cold or icilin-induced activation. These differences in human versus rodent TRPM8 antagonism by ACC-049 could arise from species differences in antibody binding and/or species specific conformational changes of TRPM8 induced by the different agonists. The antigenic peptide sequence of ACC-049 differs between human and rodents (Figure 1). ACC-049 was generated against an extracellular epitope (amino acids 917-929) close to the pore region of human TRPM8. The human peptide sequence contains a glycine in position 921 and an alanine in position 927, whereas the rodent channel contains serine residues in these positions. Different critical residues/regions within the TRPM8 channel have been described for cold, menthol and icilin (for review, see [28]). Cold activation depends on the C-terminus [29], the icilin binding site is situated within the S2-S3 linker [30], [31], and the residues important for activation by menthol are found at multiple sites, e.g., within the C-terminus, the TRP box and the S2, S4 and S4-S5 linker region of human TRPM8 [32], [33]. The specific residue differences in human versus rodent TRPM8 may account for differential pharmacology of ACC-049 at icilin versus menthol activation.

Our finding that ACC-049 blocked both human and rodent TRPM8 activation by different ligands, which bind to different regions of the channel, suggests that high affinity binding of this antibody to the pore loop locks the channel in a closed conformation. Furthermore, we showed that not only ACC-049, but also other poly- and monoclonal antibodies directed against the same pore loop blocked icilin activation of the human TRPM8 channels recombinantly expressed in CHO cells and TRPM8 channels endogenously expressed in rat DRG neurons. This suggests that there are no pharmacological differences between endogenous or recombinant TRPM8 channels for inhibition by small molecule or antibody antagonists.

These data confirm previously published findings that targeting the third extracellular loop that forms the pore in several ion channels represents a promising strategy to generate antagonist antibodies [21], [22]. Several tool antagonist antibodies to ion channels have been described including polyclonal antibodies to potassium channels, e.g. Kv1.2 and Kv3.1 [34], TRP channels, e.g. TRPC1, TRPC5, TRPV1 and TRPM3 [20], [22], [35] and CaV1.2 channels [36], as well as monoclonal antibodies to hERG [37] and TRPA1 channels [38].

While the affinity of ACC-049 and the other commercial antibodies tested in our study is not sufficient for a therapeutic consideration, our data are encouraging for two reasons. First, we observed full blockade of the agonist-induced TRPM8 activation by ACC-049, albeit at high concentrations of the antibody. In the published studies mentioned above, only partial blockade of the respective ion channels had been achieved with antibodies. For therapeutic purposes, full channel blockade may be required and our data suggest that it is possible to block channel activation fully with antibodies. Second, in single concentration experiment, we identified three monoclonal antibodies that achieved 60% to 100% blockade of icilin-induced activation of the human TRPM8 channel. Only two monoclonal antagonist antibodies, one to hERG potassium channel [37], and another to TRPA1 channel [38], have been published to date, whereas two other monoclonal antibodies against the pore loop of TRPV1 did not block channel activation [20]. Our data support the view that it is feasible to generate monoclonal antagonist antibodies that show full block of channel activation by different mechanisms. Although challenges remain, the continued research with tool antagonist antibodies, and the availability of existing and emerging technologies to improve antibody performance may enable the development of monoclonal ion channel antagonist antibody therapeutics in the future.
